# The decisive role of cardiovascular MRI delayed hyperenhancement (DHE) pattern for risk stratification for dilated cardiomyopathy

**DOI:** 10.1186/1532-429X-11-S1-P89

**Published:** 2009-01-28

**Authors:** Jose V Venero, Srinivas Murali, Mark Doyle, Vikas K Rathi, Saundra B Grant, June Yamrozik, Ronald B Williams, Diane A Vido, Geetha Rayarao, Robert WW Biederman

**Affiliations:** Allegheny General Hospital, The Gerald McGinnis Cardiovascular Institute, Pittsburgh, PA USA

**Keywords:** Dilate Cardiomyopathy, NYHA Class, Advanced Heart Failure, Event Free Survival, Improve Risk Stratification

## Introduction

Risk stratification in dilated cardiomyopathy (CMX) patients with advanced heart failure remains a growing clinical challenge. A simple manner to non-invasively risk stratify this difficult cohort would have obvious advantages.

## Hypothesis

Utilizing cardiovascular MRI (CMR), recently demonstrated to identify abnormal myocardial substrate, typically infarct or more recently infiltrative pathology via the delayed hyperenhancement technique (DHE), we hypothesize that +DHE will represent an adverse prognosis as defined by need for urgent cardiac transplantation (TX), LVAD or death in a dilated cardiomyopathy patients.

## Methods

Over 24 consecutive months, 13 patients with a dilated CMX and NYHA class III-IV heart failure underwent standard 3D CMR (1.5 T GE, Excite, Milwaukee, WI) to interrogate the pattern, distribution and extent of DHE (MultiHance, Bracco Diagnostics, Princeton, N J, USA). Patients were categorized into: 1) + DHE/+midwall Stripe 2) +DHE/-midwall Stripe and 3) -DHE/-Stripe. LVAD, Tx need, major adverse clinical events (MACE) and event free survival were evaluated over the next 6 months.

## Results

All patients were alive at 6 months for folow-up while 5 required Tx. All pts completed the CMR exam in 50 ± 10 minutes. Of 11/13 pts (85%) with +DHE, 9/11 pts (82%) had +Stripe and 2/13 pts were -DHE/-Stripe. DHE/Stripe categorization positively strongly correlated with NYHA class (r = 0.59, p < 0.05) while only weakly correlating with 3D LVEF (r = 0.14, p = ns) and EDV (r = 0.05, p = ns). However, DHE/Stripe categorization strongly predicted the need for LVAD and/or urgent Tx surgery over the ensuing 6 months (X^2^ = 5, p < 0.05). Specifically, all 5 pts requiring LVAD and/or urgent Tx by 6 months had +DHE/+Stripe while no -DHE/-Stripe pts experienced the need for LVAD or urgent Tx. Similarly, a +DHE/+Stripe strongly predicted MACE (X^2^ = 8, p < 0.005). Any +DHE pt with + *or* -Stripe, in general, predicted a more egregious course, meeting a MACE, worsening LVEF, or non-improved/worsening NYHA class. No -DHE/-Stripe patient had a major adverse clinical event, see Table 1.

**Table 1:** Table 1

DHE Status	No. of patients	Heart transplant	MACE	Unchanged/worsening NYHA	Worsening EF
**+DHE/** **+Stripe**	9	5	7	8	8
**+DHE/** **-Stripe**	2	0	1	1	1
**-DHE/** **-Stripe**	2	0	0	0	0

## Conclusion

CMX patients with advanced heart failure require an improved risk stratification policy. We believe this observation represents the first attempt to risk stratfiy systematically for those with a dilated CMX. Specifically, a simple observation of the binary nature of DHE/Stripe predicated early morbidity and mortality. Herein, using standard CMR, the presence of **+DHE/+Stripe** is highly predictive of LVAD and Tx need over the ensuing 6 months. Those **+DHE/**-**Stripe** patients have intermediate risk but *no* LVAD/Tx use, while those -**DHE/**-**Stripe** have a good prognosis. Thus, incorporating this approach into routine clinical practice may help manage CMX patients more expectantly and effectively.

